# Antipyrin in the Treatment of Seminal Emissions

**Published:** 1888-05

**Authors:** 


Antipyrin in the Treatment of Seminal Emissions.—The older remedies for this affection, camphor and lupulin, have very properly been abandoned. Kurschmann says that the sedative action of lupulin on the genital organs is far from demonstrated, and the employment of camphor is not more reliable, although Zeissl, Purjesz and others consider it the best remedy in this affection. Nux vomica, arsenic and atropine have also been recommended, while Diday prefers the bromides of potassium and sodium to all other remedies. He recommends from thirty to eighty grains of the bromide of potassium to be taken on retiring. But these large doses of bromide will produce acne, and are also liable to induce mental enfeeblement. In order to avoid the dangers of bromides, Thor, of Bucharest, has been experimenting with antipyrin in the treatment of these affections. He advises the patient to take from seven to fifteen grains of the drug on retiring. In seventeen cases, he has completely cured the complaint, without any unpleasant consequences. According to Beart, antipyrin is useful in neurasthenia of the sexual organs, but in these cases from 1 to two grains a day should be given.—*Revista de Cincias Medicas.*

